# Modelling the Variability in Immunity Build-Up and Waning Following RNA-Based Vaccination

**DOI:** 10.3390/v17121643

**Published:** 2025-12-18

**Authors:** Juan Magalang, Tyll Krueger, Joerg Galle

**Affiliations:** 1 Department of Visceral Surgery and Medicine, Inselspital, Bern University Hospital, University of Bern, Murtenstrasse 35, 3008 Bern, Switzerland; 2 Institute of Mathematical Statistics and Actuarial Science, University of Bern, Alpeneggstrasse 22, 3012 Bern, Switzerland; 3 Faculty of Information and Communication Technology, Wroclaw University of Science and Technology, Janiszewskiego 11-17, 50-372 Wrocław, Poland; tyll.krueger@pwr.edu.pl; 4 Interdisciplinary Centre for Bioinformatics (IZBI), Leipzig University, Haertelstr. 16-18, 04107 Leipzig, Germany

**Keywords:** mathematical modelling, RNA vaccines, immune response, Sobol’ indices, ODE, germinal center

## Abstract

RNA-based vaccination has been broadly applied in the COVID-19 pandemic. A characteristic of the immunization was fast-waning immunity. However, the time scale of this process varied considerably for virus subtypes and among individuals. Understanding the origin of this variability is crucial in order to improve future vaccination strategies. Here, we introduce a mathematical model of RNA-based vaccination and the kinetics of the induced immune response. In the model, antigens produced following vaccination give rise to an immune response leading to germinal center reactions and accordingly B-cell differentiation into memory B-cells and plasma cells. In a negative feedback loop, the antibodies synthesized by newly specified plasma cells shut down the germinal center reaction as well as antigen-induced differentiation of memory B-cell into plasma cells. This limits the build-up of long-lasting immunity and thus is accompanied by fast-waning immunity. The detailed data available on infection with and vaccination against SARS-CoV-2 enabled computational simulation of essential processes of the immune response. Through simulation, we analyzed to what extent a single- or double-dose vaccination provides protection against infection. We find that variability in the immune response in individuals, originating, e.g., in different immune-cell densities, results in a broad log-normal-like distribution of the vaccine-induced protection times that peaks around 100 days. Protection times decrease for virus variants with mutated antibody-binding sites or increased replication rates. Independent of these virus specifics, our simulations suggest optimal timing of a second dose about 5 weeks after the first in agreement with clinical trials.

## 1. Introduction

The immune response enables our body to defend itself against pathogens including viruses and bacteria. Mathematical models describing the kinetics of the immune response against infections with them are well established. In many cases, they combine aspects of the innate and adaptive response (e.g., in the case of influenza, [1]). While the innate response is directed against the spreading infection, the adaptive response mainly serves to protect the body from potential future infections. The effectiveness of the latter comes into focus when vaccination is applied. A core component of the adaptive response are germinal center reactions enabling the specification of cells capable of producing antibodies that can efficiently neutralize the pathogens. This process can provide lifelong protection against future infections. Modelling these reactions has attracted increasing attention in the last years [2,3].

The source of antibodies are plasma cells [4]. Two types of these cells are typically specified during immune responses against a pathogen V, namely short- and long-living plasma cells. Short-living plasma cells vanish over several weeks. They become newly induced during repeated infection with V. Their long-living counterparts are resident in the bone marrow and permanently secret V-specific antibodies. Thus, they are most efficient in suppressing repeated infections with V.

The emergence of SARS-CoV-2 led to the first-time broad application of RNA-based vaccination. Several mathematical models have been introduced that provide insight into the principles of the immune response on both infection by the virus and vaccination against it (e.g., [5]). In summer 2021, it became obvious that the immunization reached by vaccination significantly decreases in effectiveness within several months. Starting with reports about breakthrough infections from Israel [6], an increasing number of studies provided insights into this waning process [7]. Meanwhile, related effects have been integrated into mathematical models aiming at forecasts of infection numbers [8]. Some of them link the level of remaining protection and control measures such as antibody concentrations in the serum [9,10]. The cellular and molecular factors that control the individual waning of protection against infection remain largely undefined. A potential explanation of the fast waning is missing the specification of long-living plasma cells following vaccination [11].

Here, we introduce a mathematical model of RNA-based vaccination against a virus V and the kinetics of the induced immune response. We focus on the adaptive immune response and the processes leading to production of antibodies against V-specific antigens. We quantify the immunization reached by the vaccination by the concentration of the antibodies and their capability to bind the antigen. We observe waning immunity and identify potential sources of this process.

## 2. Basic Assumptions

### 2.1. Vaccination Model

We present a within-host model of RNA-based vaccination against virus V. The naive host is vaccinated with a V-specific mRNA that is encapsulated in lipid-nano-particles (LNPs). Part of the LNPs are taken up by susceptible cells (*S*)—mostly antibody-presenting-cells (APCs)—which become ‘infected’ cells (*I*). Alternatively, LNPs become degraded over time. Infected cells (*I*) produce pseudo-antigen (P), the protein encoded by the transferred RNA. The antigen triggers an adaptive immune response in the host. In order to describe this response, we extend our model in two steps. In the first step, we include the primary response comprising the GC reaction. In a second step, we add the secondary response leading to memory B-cell differentiation.

### 2.2. Primary Response Model

Initially, the presence of P leads to the activation of naïve B-cells ($B_{N}$). These cells can differentiate directly into memory B-cells ($B_{M}$) and short-living plasma cells ($B_{1}$) or are recruited into developing germinal centers (GCs), where they become germinal center B-cells ($B_{G}$) [4]. As the $B_{N}$-based $B_{M}$- and $B_{1}$-responses come with antibodies of low affinity against V and on short time scales, we consider the $B_{G}$-option only. In the GC, $B_{G}$s undergoes affinity maturation (AM), a process that, based on somatic hyper-mutation, improves the binding of their B-cell receptor (BCR) to the antigen. This process comprises expansion and the selection of $B_{G}$s. Thereby, it requires activity pertaining to APCs and specific T-cell subtypes in the GC. These details are not considered in the model. We assume a continuous increase in the average BCR affinity during V-specific AM. $B_{G}$s leave the GC, thereby differentiating either into memory B-cells ($B_{M}$), or into long-living plasma cells ($B_{2}$). These cells enrich in subcapsular proliferative foci (SPF) [12] and the bone marrow [13], respectively.

### 2.3. Secondary Response

In the case of a prolonged presence of pseudo-antigen or repeated vaccination, $B_{M}$-cells that have developed in the GCs and moved into SPFs become activated by the antigen presented by subcapsular sinus macrophages (SSMs) [12]. Subsequently, they amplify and differentiate into short-living plasma cells ($B_{1}$) [14]. In part, $B_{M}$-cells re-enter GCs for further maturation. Here, we neglect to explore this option. $B_{1}$- and $B_{2}$-cells produce antibodies (A) of different affinities that help in fighting V infection. Waning of the vaccine-induced B-cell responses is analyzed studying the time course of antibody affinity and concentration.

**Figure 1:**
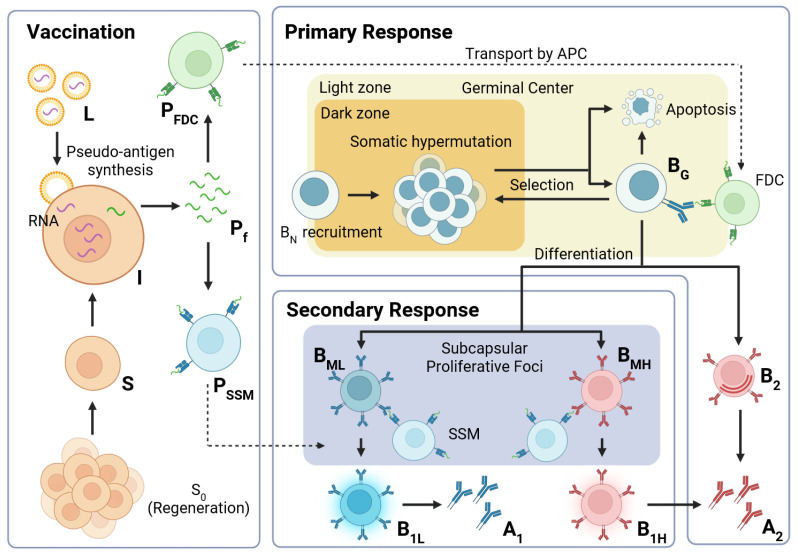
Model schema. Vaccination, followed by production of pseudo-antigen P, induces (i) a primary response (activation of GC, affinity maturation (AM) and specification of $B_{M}$- and $B_{2}$-cells) and (ii) a secondary response (activation of $B_{M}$ cells, specification of $B_{1}$-cells). Both contribute to the production of antibodies that help fight future infection. The model introduced does not comprise details regarding the transport of antigen and the AM.

Parameters of our vaccination and immune response model are derived mainly from studies on RNA-based vaccination against SARS-CoV-2. Thus, in the following sections, spike protein refers to the SARS-CoV-2 variants of this protein. Most of the related studies focus on the Wuhan variant. Protection against other variants can be simulated, e.g., considering the changed antibody–antigen-binding affinity [15]. Some parameters of the model are taken from animal studies (indicated). A schematic of the entire model i.e., the extended model, is provided in Figure 1.

## 3. Mathematical Formulation: Vaccination Model

We describe vaccination as a systemic ‘infection’ with LNPs. Starting at the injection site, LNPs distribute passively throughout the body in a few hours [16]. We neglect differences between tissues and take blood concentration as the mean (fast diffusion limit). Mainly, APCs uptake LNPs and produce pseudo-antigen [17]. We assume that these antigens are distributed passively throughout the body, and their concentration blood represents the mean concentration of free-floating antigens. However, part of the antigen is actively transported by immune cells to lymph nodes, leading to high local concentrations within developing germinal centers (GCs) and subcapsular proliferative foci (SPF).

### 3.1. LNPs (L)

In the vaccine, RNA is encapsulated in LNPs. Each LNP contains about 5 RNA molecules (1–10, [18]). In the body, the concentration of *L* decreases due to LNP uptake via endocytosis by susceptible cells (*S*) and LNP degradation.

(1) $$\frac{dL}{dt}=-k_{L}SL-\frac{L}{\tau_{L}}.$$

Here, $k_{L}$ is the LNP uptake rate by susceptible cells. We estimate $k_{L}$ from decay rates of spike-encoding RNA in blood plasma [19]. Assuming a constant density, $S=S_{0}$, these decay rates suggest an RNA uptake rate larger than $6.9\times 10^{-7}\mathrm{mL}/\left(\mathrm{day}\times \mathrm{cell}\right)$. Considering 5 RNA molecules per LNP, we assume: $k_{L}=2.0\times 10^{-7}\mathrm{mL}/\left(\mathrm{day}\times \mathrm{cell}\right)$.

Free RNA is degraded in blood plasma within hours. LNPs protect RNA from degradation. Thus, RNA degradation immediately follows LNP degradation. We set the lifetime of LNPs in the body equal to the lifetime of encapsulated RNA, $\tau_{L}=7$ days [19]. This is a long lifetime for LNPs. Nevertheless, it is supported by experiments detecting Spike-RNA up to 28 days after vaccination [20].

### 3.2. Susceptible Cells (S)

LNPs are taken up mostly by APCs including dendritic cells and macrophages (primates, [16,17]). We assume that susceptible cells (*S*) that uptake LNPs become ‘infected’ cells (*I*) i.e., they downregulate endocytosis and start synthesis of the RNA-encoded protein. APCs downregulate endocytosis following maturation induced by pathogens [21]. In the case of LNPs, PEG (poly-ethylene-glycol) acts as pathogen [19]. Accordingly, the density S decreases in the presence of LNPs and of free-floating antigen ($P_{f}$) secreted by infected cells (see below). *S* increases if naïve APCs travel to the vaccination site. In our model, this process is described as cell-inflow from an external pool that ensures that the initial density of susceptible cells $S_{0}$ is restored. Thus, changes in *S* are described as:

(2) $$\frac{dS}{dt}=-k_{S}LS-k_{APC}P_{f}S+k_{1}\left(S_{0}-S\right).$$

Here, $k_{S}$ is the infection rate of susceptible cells by LNPs, i.e., the rate of switching a susceptible cell into a pseudo-antigen producing cell. A successful switch comprises, besides endocytosis of LNPs, RNA endosomal escape [22]. We calculated $k_{S}$ assuming that $n_{L}\geq 1$ LNPs are required to infect one cell ($k_{L}/k_{S}=n_{L}$ copies/cell). $k_{APC}$ is the activation rate of susceptible cells per captured antigen (see below). Susceptible cells are recruited from a cell pool that has a constant density $S_{0}$. We estimated it from the density of tissue macrophages in muscle ($10^{6}$ cells/mL, [23]). The rate of recruitment $k_{1}$ was set to $1/14\;{\mathrm{days}}^{-1}$, consistent with long-lasting regeneration of APC pools after SARS-CoV-2 infection [24].

### 3.3. Infected Cells (I)

Infected cells start the synthesis of pseudo-antigen (P, see below), the protein encoded by the transferred RNA. The density of *I* changes as follows:

(3) $$\frac{dI}{dt}=k_{S}SL-\frac{1}{\tau_{I}}I.$$

Here, $\tau_{I}$ is the lifetime of infected cells. We assume $\tau_{I}=4$ days, which is the lifetime of circulating dendritic cells [25]. This time is much shorter than the lifetime of follicular dendritic cells (FDC) presenting antigen in the GC (see below), which is in mice larger than two weeks [26].

### 3.4. Pseudo-Antigen P

Antigen synthesized by infected cells (*I*) is released as free-floating protein ($P_{f}$) into body fluids [27]. We assume that susceptible cells (*S*) capture the protein, become activated, move into lymph nodes, and relay it to FDCs in the GC or to SSMs in the SPF (for the sake of simplicity in equal amounts). These cells present it, in the form of immune complexes $P_{FDC}$ or $P_{SSM}$ to germinal center B-cells ($B_{G}$) and memory B-cells ($B_{M}$), respectively. We do not model the antigen transport into lymph nodes (see e.g., mice studies on $B_{G}$ cells by Heesters et al. [26]). The free-floating protein ($P_{f}$) and the immune complexes ($P_{FDC}$, $P_{SSM}$) change as follows:

(4) $$\frac{dP_{f}}{dt}=p_{P}I-k_{p}SP_{f}-\frac{P_{f}}{\tau_{Pf}},$$

(5A,B) $$\frac{dP_{FDC}}{dt}=\frac{k_{P}}{2}SP_{f}-\frac{P_{FDC}}{\tau_{FDC}},\;\frac{dP_{SSM}}{dt}=\frac{k_{P}}{2}SP_{f}-\frac{P_{SSM}}{\tau_{SSM}}.$$

The production rate was set to: $p_{P}=25\;\mathrm{copies}/\left(\mathrm{day}\times \mathrm{cell}\right)$ in agreement with experiments (baby hamster kidney cells, [28]). The lifetime of the free-floating protein $\tau_{Pf}$ was set to 2 days. Under this setting, protein concentration reaches baseline 1–2 weeks after vaccination in agreement with Cognetti et al. [29]. $P_{FDC}$ can survive for long periods of time [26]. We set its lifetime, $\tau_{FDC}$, to 20 days. Assuming that it can be released again in small amounts might explain why the spike protein remains detectable in blood for more than 10 weeks [30]. There is no report about similar behavior for $P_{SSM}$. Thus, we assume: $\tau_{SSM}=\tau_{Pf}$. We consider degradation to represent the dominant process responsible for loss of free-floating protein. Thus, for the rate of $P_{f}$ capture by *S*-cells should hold: $k_{P}S\ll 1/\tau_{P}f$. This relation is ensured for $S\leq S_{0}$ setting: $k_{P}=1.0\times 10^{-7}\mathrm{mL}/\left(\mathrm{day}\times \mathrm{cell}\right)$. We calculated $k_{APC}$ assuming that one antigen activates one cell ($k_{P}/k_{APC}=1$ copy/cell). This implicates the strongest possible effect on S (see Equation (2), single-hit viral infection kinetics). Effects seen for lower $k_{APC}$ are discussed in the appendices.

### 3.5. Injected LPC Density

According to Kent et al. [19], the measurable concentration of total RNA peaks in the peripheral blood at day 1 after vaccination at concentrations between $10^{5}$ and $10^{6}$ copies/mL. To cope with these findings, we set: $L_{0}=2.5\times 10^{5}\;n_{L}$ copies/mL. Thus, for lower infection rates, higher numbers of RNA molecules have to be provided by vaccination, keeping $L_{0}k_{S}=0.05$ copies/(cell × day) fixed. Notably, $L_{0}$-scaling for fixed $L_{0}k_{S}$ affects only the density of *L* (see Appendix A).

### 3.6. Second Vaccination Time

Our simulations start with the first dose of the vaccine $L_{0}$ at t=0. Let $t^{*}>0$ be a time at which a second dose of the vaccine is administered. Such a subsequent dose of vaccines is simulated as an increase in *L* by the value $L_{0}$ at time $t^{*}$.

### 3.7. Analytical Solutions

**Figure 2:**
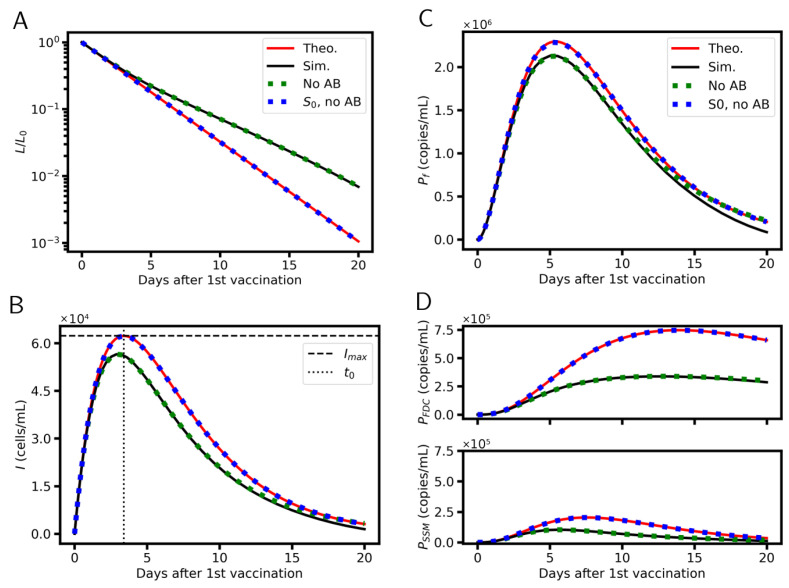
Time course of the concentration of: (**A**) free LNPs ($L/L_{0}$), (**B**) infected cells (I), (**C**) free-floating protein ($P_{f}$) and (**D**) interaction complexes ($P_{FDC}$, $P_{SSM}$) after a single vaccination. Those shown are analytical (red) and numerical solutions (black lines and symbols). The maximum density of infected cells $I_{max}$ and their time point after vaccination $t_{0}$ are specified in the Appendix A.

The vaccination model can be solved analytically if *S* is kept constant; $S=S_{0}$ (Figure 2). The equations are given in the Appendix A. For the LNP concentration (*L*) one obtains an exponential decay. The density of infected cells (*I*) shows a single peak. For the reference model, the peak is reached after about 3 days. The concentrations of free-floating protein ($P_{f}$) and of interaction complexes ($P_{SSM}$, $P_{FDC}$) peak a few days after. For the reference parameter set, the maximum concentration of $P_{f}$ is reached after about 5 days in agreement with Ogata et al. [27]. According to our settings, $P_{SSM}$ peaks at the same time, and $P_{FDC}$ after about 14 days.

We compared the analytical with numerical solutions of the extended model described below, and with numerical solutions of the extended model without antibodies (no AB) and without antibodies at constant $S=S_{0}$ ($S_{0}$, no AB). We found that (i) newly synthesized antibodies do not affect the behavior before day 13 (see deviation in $P_{f}$) and (ii) variable S has a large impact on $P_{FDC}$ and $P_{SSM}$ (Figure 2).

Thus, in order to analyze the early dynamics of antigen production following RNA-based vaccination, the immune response can be neglected. A sophisticated model of the pool of susceptible cells, however, might be required in order to model the B-cell response (depending on $P_{FDC}$ and $P_{SSM}$) quantitatively. Here, we proceed with our simplified model.

## 4. Mathematical Formulation: Primary Response Model

### 4.1. Naïve B-Cell $\left(B_{N}\right)$

Activation of an adaptive immune response requires, in addition to the presence of antigen, the presence of naïve B-cells ($B_{N}$) which can become activated. Naïve B-cells account for about 50% of the B-cells in adults [14]. Due to their fast turnover, we consider a constant pool of $B_{N}$ (steady state). As B-cells represent about 10% of the peripheral-blood mononuclear cells [31], the $B_{N}$ density can be estimated as $B_{N}=5\times 10^{4}$ cells/mL.

### 4.2. Germinal Center B-Cells $\left(B_{G}\right)$

If APCs present antigen within the GC ($P_{FDC}>0$), naïve B-cells ($B_{N}$) become activated and are recruited to become $B_{G}$s. They start processes of expansion, selection, and maturation. Depending on the details of the AM process, they differentiate after several days into memory B-cells ($B_{M}$) or long-living plasma cells ($B_{2}$). The GC reaction can take up to 14 weeks [32]. It includes internalization of antigen ($P_{FDC}$) by $B_{G}$ cells and thus consumes $P_{FDC}$. We neglect this process in our model. The density of $B_{G}$ changes as follows:

(6) $$\frac{dB_{G}}{dt}=c_{N}g_{GC}B_{N}+\left(c_{0}-a_{pD}-\epsilon \right)B_{G}-a_{pL}\left(1-g_{GC}\right)B_{G}.$$

Here, $c_{N}$ is the maximum recruitment rate of $B_{N}$ into GCs. With $c_{N}=10^{-3}{\mathrm{day}}^{-1}$, the maximum recruitment per day reaches 50 cells/mL. With one GC per ml (see below), this gives 50 cells per GC as suggested by Robert et al. [33].

Recruitment is activated by immune complexes ($P_{FDC}$). $B_{G}$ cells bind $P_{FDC}$ via their BCR. Thereby, they compete with free-floating antibodies A (see below). Thus, the strength of GC activation $g_{GC}$ depends on the concentrations of immune complexes $P_{FDC}$ and of the antibodies. Details are provided in the Appendix B.

According to Mayer et al. [34], cell amplification ($c_{0}$) balances the cell apoptosis ($a_{pD}$) in the active GC. Indeed, in mice, a rate of $B_{G}$-amplification: $c_{0}=2.0\;{\mathrm{day}}^{-1}$ was found [35], being in the same range as the apoptosis rate $a_{pD}=2.0\;{\mathrm{day}}^{-1}$ [34]. Thus, we assume $T=\left(c_{0}-a_{pD}\right)B_{G}=0$. The rate ϵ of cells leaving GC is small. Data by Robert et al. [33] suggest an upper limit of $0.15\;{\mathrm{day}}^{-1}$. We set $\epsilon =0.05\;{\mathrm{day}}^{-1}$. The rate $a_{pL}$ denotes the maximum apoptosis rate in the inactive GC. We set $a_{pL}=0.5\;{\mathrm{day}}^{-1}$, ensuing a fast shutdown of the GC if activation $g_{GC}$ decreases. For T=0, Equation (6) simplifies to:

(6^^#^^) $$\frac{dB_{G}}{dt}=c_{N}g_{GC}B_{N}-\epsilon B_{G}-a_{pL}\left(1-g_{GC}\right)B_{G}.$$

Thus, for $g_{GC}=1$ (maximum activation), a maximum density of $B_{G}=\left(c_{N}/\epsilon \right)B_{N}=10^{3}$ cells/mL can be reached. These cells are confined in the volume of all GCs that supply one mL of body fluid. For vaccination into the deltoid muscle, only axillary lymph nodes might be involved in the immune response (20–49 nodes, [32]). Thus, assuming (i) that about 10% of the 600 lymph nodes in humans become activated during vaccination, and (ii) 100 GCs per lymph nodes as found in macaques [36], an average tissue density of about one V-induced GC per ml can be estimated. Thus, for a GC volume of $2.0\times 10^{-5}$ mL [37], the local density of $B_{G}$ can reach $5.0\times 10^{7}$ cells/mL (see also: Appendix B).

### 4.3. Affinity Maturation

The activation of the GC reaction and ongoing selection during this reaction depend on the affinity of the BCR of $B_{N}$- and $B_{G}$-cells for the antigen, respectively [38]. Affinity is commonly defined as $1/K_{D}$, where $K_{D}$ is the dissociation constant of the reaction of interest. For BCR–antigen complex formation, $K_{D}$ is typically larger than nanomolar [39]. We assume a minimal affinity $a_{BG}^{0}$, which is required for $B_{N}$ recruitment to the GC. Thus, $B_{G}$s have an affinity:

(7) $$a_{BG}=a_{BG}^{0}A_{BCR},$$

with $A_{BCR}\geq 1$. In the model, only ratios of affinities are relevant (see Appendix B). Thus, $a_{BG}^{0}$ can have arbitrary values. We set $A_{BCR}^{min}=1$ for recruited $B_{N}$ cells and assume that in course of AM, $A_{BCR}$; thus, the BCR affinity increases as follows:

(8) $$\frac{dA_{BCR}}{dt}=a_{ff}+w\left(A_{BCR}^{min}-A_{BCR}\right)=w\left(A_{BCR}^{max}-A_{BCR}\right)$$

The maturation process increases $A_{BCR}$ by a constant value $a_{ff}$ per day. The second term estimates the loss of affinity of the GC cells due to the fact, that cells with low BCR affinity ($A_{BCR}^{min}$) are recruited and cells with higher affinity ($A_{BCR}$) leave the GC. We set *w* equal to the fraction of cells that leave the GC, $w=\epsilon =0.05\;{\mathrm{day}}^{-1}$. The maximum $A_{BCR}^{max}=A_{BCR}^{min}+\left(a_{ff}/w\right)$ can be calculated for a given $a_{ff}$. It depends on the mutation frequency, which is determined by T-cell help [40]. We set $a_{ff}=1.45$ per day and accordingly $A_{BCR}^{max}=30$. For vanishing V-specific maturation ($a_{ff}=0$), $A_{BCR}$ reduces again to $A_{BCR}^{min}$ (see below).

### 4.4. $B_{G}$ Specification

$B_{G}$ cells specify into $B_{M}$- and $B_{2}$-cells [41]. We consider two types of $B_{M}$-cells, $B_{ML}$ and $B_{MH}$, with low ($a_{BML}=a_{BG}^{0}A_{BCR}^{min}$) and high ($a_{BMH}=a_{BG}^{0}A_{BCR}^{max}$) affinity, respectively. All $B_{2}$-cells have high BCR affinity ($a_{B2}=a_{BG}^{0}A_{BCR}^{max}$). The fractions of $B_{G}$-cells that differentiate into $B_{ML}$-, $B_{MH}$-, and $B_{2}$-cells is chosen such that the mean value $a_{BG}$ is conserved [4].

### 4.5. Memory B-Cells $\left(B_{M}\right)$

Initially, the major fraction of amplifying $B_{G}$-cells differentiate into $B_{ML}$-cells. With progressing AM, i.e., increasing $A_{BCR}$, more and more $B_{MH}$-cells are specified. The density of $B_{ML}$- and $B_{MH}$-cells evolves as follows:

(9) $$\frac{dB_{ML}}{dt}=f\epsilon B_{G}-\frac{B_{ML}}{\tau_{BM}},\;f=\frac{A_{BCR}^{max}-A_{BCR}}{A_{BCR}^{max}-A_{BCR}^{min}},$$

(10) $$\frac{dB_{MH}}{dt}=v\left(1-f\right)\epsilon B_{G}-\frac{B_{MH}}{\tau_{BM}}.$$

The lifetime of $B_{M}$-cells, $\tau_{BM}$, was set to 18 days, as observed for elderly [42]. *v* is the fraction of high-affinity $B_{G}$-cells that differentiate into $B_{MH}$-cells, while a fraction of (1−v) differentiate into $B_{2}$- (plasma) cells. In mice, 10 days after immunization, plasma cells nearly exclusively carry high-affinity BCRs and represent less than 3 percent of all cells with high-affinity BCRs [43]. Setting: v=0.9; similar properties are observed at maximum response around day 21 (single dose; see results).

### 4.6. Long-Living Plasma Cells $\left(B_{2}\right)$

$B_{2}$ specification requires fast and repeated cycling in the active GC [44]. This might be enabled by strong T-cell help, which is triggered by particularly high BCR affinity. We assume a stochastic nature of the process. In the model, a small fraction (1−v) of high-affinity $B_{G}$-cells becomes $B_{2}$-cells if the GC matures.

(11) $$\frac{dB_{2}}{dt}=\left(1-v\right)\left(1-f\right)\epsilon B_{G}-\frac{B_{2}}{\tau_{B2}}.$$

The lifetime of $B_{2}$-cells, $\tau_{B2L}$, was set to $\tau_{BML}=180$ days (minimum in mice: 90 days [45]). $B_{2}$-cells permanently secrete high-affinity antibodies $A_{2}$ (see below).

## 5. Mathematical Formulation: Secondary Response Model

In a secondary response to antigen, $B_{M}$-cells located in SPFs become activated, expand, and differentiate into $B_{1}$-cells that produce antibodies capable of neutralizing antigen and labeling infected cells for lysis. $B_{M}$ activation depends on the antigen affinity of their BCR [4]. We neglect antigen consumption by $B_{M}$-cell activation.

### 5.1. Expanding Memory B-Cells $\left(B_{M}\right)$

If activated, memory B-cells ($B_{M}$) are capable of fast amplification and differentiation into short-living plasma cells ($B_{1}$) [46]. Activation is triggered by $P_{SSM}$. $B_{M}$-cells bind the antigen via their BCR. Thereby, they compete with free-floating antibodies. Under such conditions, the densities of $B_{ML}$ and $B_{MH}$ cells evolve as follows:

(9^#^) $$\frac{dB_{ML}}{dt}=f\epsilon B_{G}+\left(1-h\right)c_{1}g_{BML}B_{ML}-\frac{B_{ML}}{\tau_{BM}},$$

(10^#^) $$\frac{dB_{MH}}{dt}=v\left(1-f\right)\epsilon B_{G}+\left(1-h\right)c_{1}g_{BMH}B_{MH}-\frac{B_{MH}}{\tau_{BM}}.$$

The strength of $B_{M}$ activation $g_{BML}$ and $g_{BMH}$ depends on the concentrations of $P_{SSM}$ and of the antibodies $A_{1}$ and $A_{2}$. Details are given in Appendix B. The maximum amplification rate for $B_{M}$-cells, $c_{1}$, is set to 0.6 ${\mathrm{day}}^{-1}$ (mice, at maximum stimulation 3 divisions in 5 days on average, [46]). The parameter *h* quantifies the part of amplifying cells that specify into $B_{1}$-cells. We set h=0.6.

### 5.2. Short-Living Plasma Cells $\left(B_{1}\right)$

Short-living plasma cells are derived from activated memory B-cells ($B_{M}$). Compared to long-living plasma cells ($B_{2}$), they have a relatively short lifetime of a few days. Their density evolves as:

(12) $$\frac{dB_{1L}}{dt}=hc_{1}g_{BM}B_{ML}-\frac{B_{1L}}{\tau_{B1}}$$

(13) $$\frac{dB_{1H}}{dt}=hc_{1}g_{BM}B_{MH}-\frac{B_{1H}}{\tau_{B1}}$$

We set the lifetime of $B_{1}$-cells to $\tau_{B1}=5$ days [47]. $B_{1L}$ ($B_{1H}$) cells permanently secrete the low- (high-) affinity antibodies $A_{1}$ and $A_{2}$, respectively (see below).

### 5.3. Antibodies (A)

Antibodies with low ($A_{1}$) and high ($A_{2}$) affinity are produced by $B_{1L}$-plasma cells and by $B_{1H}$- and $B_{2}$-plasma cells, respectively. As the antibodies have different antigen affinity, we describe them independently. Their turnover can be described as:

(14) $$\frac{dA_{1}}{dt}=p_{A}B_{1L}-\frac{A_{1}}{\tau_{A}}$$

(15) $$\frac{dA_{2}}{dt}=p_{A}\left(B_{1H}+B_{2}\right)-\frac{A_{2}}{\tau_{A}}$$

where $\tau_{A}=60$ days denotes the lifetime of antibodies (30 days [48], 90 days, [49]) and $p_{A}=2.0\;\mathrm{ng}/\left(\mathrm{day}\times \mathrm{cell}\right)$ is the antibody production rate (0.8 ng/(day×cell) spleen, 3.4 ng/(day×cell) peripheral blood [50]).

We assume that following plasma cell differentiation, antibody affinity matches BCR affinity of the cell of origin. Nevertheless, association constants with antigen might differ between surface-bound BCR and antibodies, e.g., due to steric hindrance [51]. Details are given in the Appendix B.

Protection of the host against virus by antibodies A is at least twofold: Antibodies mark infected cells for destruction by antibody-dependent cell-mediated cytotoxicity (ADCC), antibody-dependent cellular phagocytosis (ADCP) or antibody-dependent complement deposition (ADCD) [52]. We assume that all antibodies have this labeling capacity. In addition to labeling cells for destruction, antibodies can directly neutralize antigen, which here means binding $P_{f}$ and suppressing its functionality. Assuming that all antibodies have both capabilities and distribute throughout the body (fast diffusion limit), we modify Equations (3), (4), (14) and (15) to:

(3^#^) $$\frac{dI}{dt}=k_{S}SL-\frac{1}{\tau_{I}}I-\left(\beta_{IA1}A_{1}+\beta_{IA2}A_{2}\right)I,$$

(4^#^) $$\frac{dP_{f}}{dt}=p_{P}I-k_{p}SP_{f}-\frac{P_{f}}{\tau_{Pf}}-\left(\beta_{PfA1}A_{1}+\beta_{PfA2}A_{2}\right)P_{f},$$

(14^#^) $$\frac{dA_{1}}{dt}=p_{A}B_{1L}-\frac{A_{1}}{\tau_{A}}-\left(\gamma_{IA1}I+\gamma_{PfA1}P_{f}\right)A_{1},$$

(15^#^) $$\frac{dA_{2}}{dt}=p_{A}\left(B_{1H}+B_{2}\right)-\frac{A_{1}}{\tau_{A}}-\left(\gamma_{IA2}I+\gamma_{PfA2}P_{f}\right)A_{2}.$$

Here, $\beta_{IA1}$ and $\beta_{IA2}$ are the rates of cell destruction and $\beta_{PfA1}$ and $\beta_{PfA2}$ the rates of antigen neutralization induced by $A_{1}$ and $A_{2}$, respectively. These processes consume antibodies. $\gamma_{IA1}$, $\gamma_{IA2}$, $\gamma_{PfA1}$ and $\gamma_{PfA2}$ are the rates of consumption per infected cell *I* and pseudo-antigen $P_{f}$, respectively. Details are provided in the Appendix B.

### 5.4. Antibody-Dependent Feedback on AM

In negative feedback, antibodies control the activity of the GC (see Equation (6^#^)). We assume that they enter the GC and bind the antigen in competition with $B_{G}$-cell BCRs [53]. This also dampens or even eliminates AM towards dominant antigen V [33]. For $A_{BCR}$, it follows:

(8^#^) $$\frac{dA_{BCR}}{dt}=v\left(A_{BCR}^{min}-A_{BCR}+\left(A_{BCR}^{max}-A_{BCR}^{min}\right)g_{GC}\right)$$

Thus, Equation (8) yields for $g_{GC}=1$ only. For $g_{GC}=0$, $A_{BCR}$ is forced to decrease to $A_{BCR}^{min}$. Consequently, antigen-specific AM is terminated at the time point where $P_{f}$ is successfully degraded and/or the antibody titer increases. In vivo, the GC reaction can be still active in case the AM target switches to subdominant antigen [54]. We simulate only dominant antigen-specific AM; thus, GC lifetime is shorter than that seen during GC reaction in response to complex antigen.

Notably, the permanent inflow of non-specific BN has been observed in mice and was considered to result in low affinity of $B_{G}$ cells in late GCs [55,56]. This suggests that due to the AM towards non-dominant antigen, initial selection towards dominant antigen ($A_{BCR}^{min}=1$) vanishes and values $A_{BCR}^{min}<1$ could occur. We neglect this effect.

## 6. Simulation Results

### 6.1. Systems Dynamics

**Figure 3:**
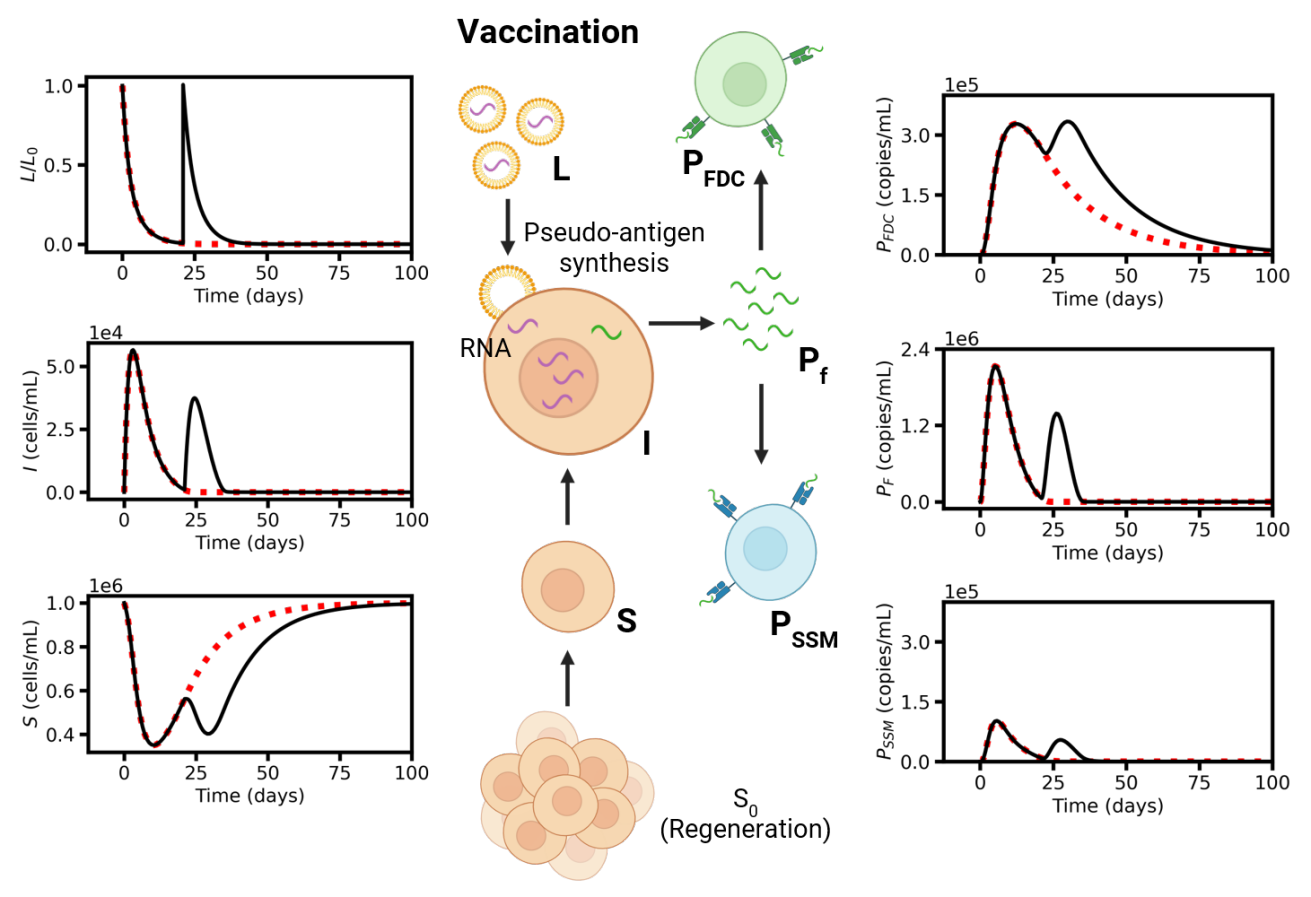
Model dynamics I. Simulation of single- (day 0, red dotted line) and double-dose vaccination (day 0 and day 21, black solid line) for the reference parameter set. Vaccination model: Concentration of LNPs (*L*), infected cells (*I*), susceptible cells (*S*), free-floating protein ($P_{f}$), and the two types of immune complexes ($P_{FDC}$, $P_{SSM}$).

**Figure 4:**
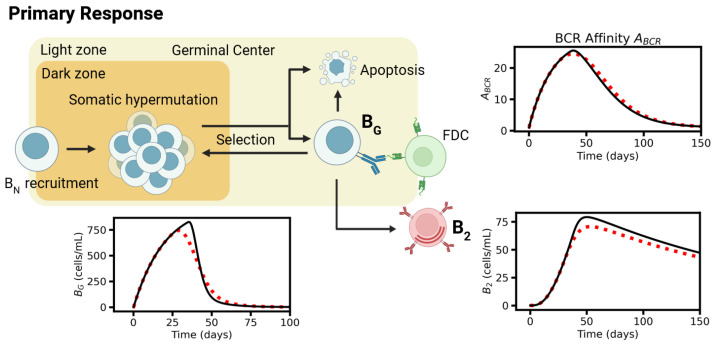
Model dynamics II. Simulation of single- (day 0, red dotted line) and double-dose vaccination (day 0 and day 21, black solid line) for the reference parameter set. Primary response model: Density of GC B-cells ($B_{G}$), the BCR affinity of $B_{G}$ cells ($A_{BCR}$), and the density of the long-living plasma cells ($B_{2}$).

**Figure 5:**
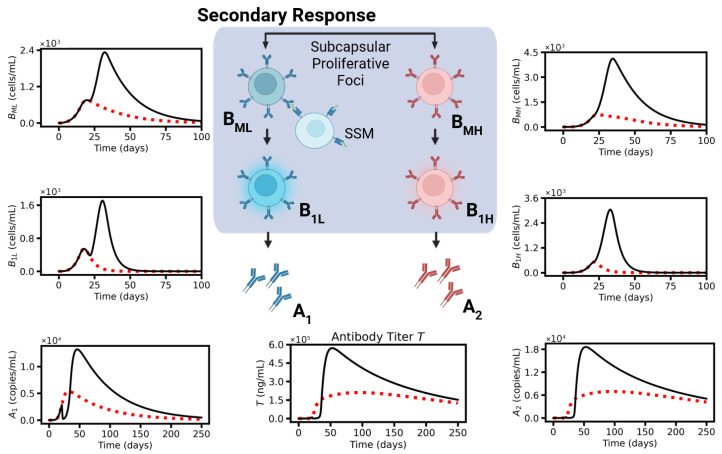
Model dynamics III. Simulation of single- (day 0, red dotted line) and double-dose vaccination (day 0 and day 21, black solid line) for the reference parameter set. Secondary response model: Low- and high-affinity memory B-cells ($B_{ML}$, $B_{MH}$), low- and high-affinity short-living plasma cells ($B_{1L}$, $B_{1H}$). Protective potential: Low- ($A_{1}$) and high- ($A_{2}$) affinity antibody concentration and the resulting titer T.

The extended model can be only solved numerically. We started analyzing basic model properties, applying the reference parameter set provided in Table A1, Table A2, Table A3 and Table A4. Parameters that were not available from experiments were set such that, the model reproduces properties of the peripheral blood following vaccination against SARS-CoV-2. We compared the response to single- and double-dose vaccination (Figure 3, Figure 4 and Figure 5). The first dose stimulates antigen production that peaks a few days later (compare Figure 2). At the time point of the second dose, the concentrations of LNPs (*L*) and infected cells (*I*) but also of pseudo-antigen $P_{f}$ and $P_{SSM}$ have already dropped close to zero (Figure 3). The second dose massively increases these concentrations again but has a rather small effect on $P_{FDC}$. Accordingly, the primary response, the GC reaction, is similar for both single- and double-dose vaccination; in particular, similar numbers of $B_{2}$ cells are induced (Figure 4). Thus, a second dose does not improve long-term protection very much.

The second dose controls short-term protection via amplification of the $B_{M}$-$B_{1}$ response, i.e., amplification of the secondary response. A single-dose vaccination results in a peak concentration of $P_{f}$ of about 1 to $2\times 10^{6}$ copies/mL in agreement with spike protein peaks (68 pg/mL, [27]). This reaction elicits a weak secondary $B_{M}$-$B_{1}$ response only (Figure 5). Stronger response requires repeated $B_{M}$ activation and amplification. This is achieved by a second dose, which stimulates production of new antigen. Massive $B_{1}$ induction results in much higher antibody titers. However, these amplified titers remain for less than 200 days only. Notably, the simulated peak density of $B_{M}$-cells after the second dose reaches $10^{3}$–$10^{4}$ cells/mL and that of antibodies 5 to $10\times 10^{4}$ ng/mL. Both results are in agreement with concentrations found by Goel et al. [57]. Moreover, $B_{1}$ cell densities peak between week 4 and 5 after the first vaccination in agreement with Turner et al. [32].

As shown in Figure 3, Figure 4 and Figure 5, the systems dynamics recapitulate many aspects of RNA vaccination against SARS-CoV-2. About 2 weeks after the 2nd vaccination, the immune response provides high amounts of antibodies that can help in fighting a future viral infection. While the feedback of these antibodies was negligible for the infection scenario following the first vaccination (first 2 weeks, Figure 2), we then assessed its impact on the whole B-cell response.

**Figure 6:**
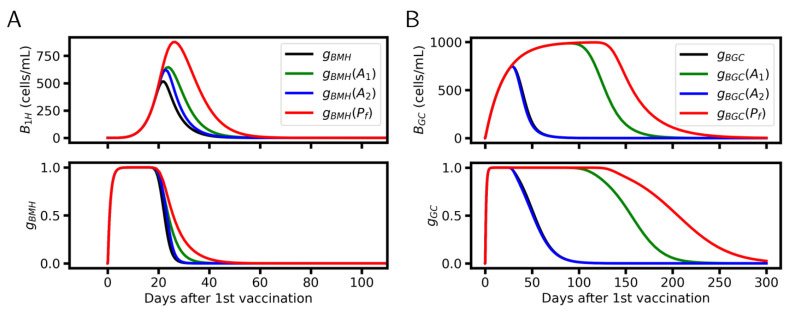
Antibody feedback after a double-dose vaccination (reference parameter set). (**A**) Density of $B_{1H}$-cells (**upper row**). The emergence of low affinity antibodies is sufficient to stop $B_{1H}$-specification. $B_{1H}$ peaks at the point, where the activation function $g_{BM}$ (**lower row**) starts decreasing. Compared is the behavior for full feedback ($g_{BM}$) with that based on $A_{1}$ ($g_{BM}\left(A_{1}\right)$) or $A_{2}$ ($g_{BM}\left(A_{2}\right)$) only and the behavior without feedback ($g_{BM}\left(P_{f}\right)$). (**B**) Density of $B_{G}$-cells (**upper row**). The shutdown of the GC is triggered by the emergence of $A_{2}$-antibodies. $B_{G}$ peaks at the point, where the activation function $g_{GC}$ (**lower row**) starts decreasing. Compared is the behavior for full feedback ($g_{GC}$) with that based on $A_{1}$ ($g_{GC}\left(A_{1}\right)$) or $A_{2}$ ($g_{GC}\left(A_{2}\right)$) only and the behavior without feedback ($g_{GC}\left(P_{f}\right)$).

Detailed analysis revealed that antibody feedback has large impact on plasma cell specification (Figure 6). The emergence of antibodies represses $B_{M}$ activation by competitive $P_{SSM}$-binding with $B_{M}$-BCRs (Figure 6A). Without antibody feedback, the concentration of the $B_{1H}$-maximum would double and arise about 10 days later. To keep it at about 22 days, $A_{1}$-feedback is sufficient. Shutdown of the GC is controlled by $A_{2}$-feedback (Figure 6B), i.e., by competitive $P_{FDC}$-binding of these antibodies with the $B_{G}$-BCRs. Thereby, increasing $A_{2}$ concentration limits GC lifetime to about 50 days and thus keeps $B_{2}$ specification low. Without antibody feedback, the GC would remain activated up to 200 days.

### 6.2. Protective Potential of Vaccination

The immune response induced by vaccination can potentially protect the individual against infection with virus V. Whether or not protection after some time is given is commonly estimated by the remaining antibody titer. Due to inter-individual differences of the infection, the critical titer required to prevent infection varies as well.

Here, we used a model of natural infection to calculate the critical titer $T_{c}$ depending on properties of the virus and the individual immune response. For details, we refer to Appendix C. We measure the titer in equivalents of low-affinity antibodies $A_{1}$ in ng/mL. If it is $T_{c}\leq 0$ ng/mL, the infection can be controlled without antibodies. Otherwise, antibodies are required. As both $A_{1}$ and $A_{2}$ antibodies contribute to protection, the time-dependent titer acquired by vaccination is given by:

(16) $$T=A_{1}A_{BCR}^{min}+A_{2}A_{BCR}^{max}\;\left[\mathrm{ng}/\mathrm{mL}\right]$$

If *T* is larger than the critical titer $T_{c}$, the infection does not spread. Otherwise, it will spread until the immune response on the infection controls it. We analyzed whether a double vaccination as described by our model provides the required level of protection and how long it lasts.

**Figure 7:**
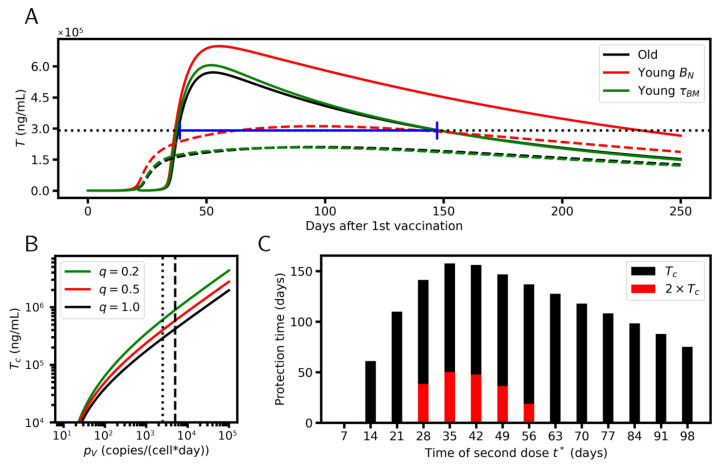
Protection level. (**A**) Simulation results for the titer after a single dose (dashed lines) and a double dose ($t^{*}=3$ weeks, solid lines) vaccination. The response after a double-dose vaccination provides protection against infection (dotted line: $T_{c}$) for about 15 weeks (blue label). Accounting for properties of young individuals can increase the protection time. (**B**) The critical titer increases with increasing virus replication rate $p_{V}$ and decreasing antibody–antigen affinities (achieved by multiplying them with a factor of *q*). The dotted line refers to the reference value of $p_{V}$, dashed line to twice of $p_{V}$. (**C**) Protection time vs time to second dose after first dose ($t^{*}$), comparing the reference critical titer $T_{c}$ (black) and twice of $T_{c}$ (red). A maximum is seen at $t^{*}=5$ weeks.

Figure 7A shows simulation results for the titer applying the reference parameter set. The critical titer $T_{c}$ was calculated as described in the Appendix C applying the infection parameter set provided in Table A5. After the first dose, $T_{c}$ is not reached and no protection is achieved. The second dose ($t^{*}=3$ weeks) increases the titer above $T_{c}$ by a considerable amount. This is sufficient to provide protection between day 37 and 143 after first vaccination, i.e., for about 15 weeks.

Notably, we derived part of the reference parameter set from data that are representative of the blood of the elderly. Examples are the lifetime of $B_{M}$-cells, $\tau_{BM}=18$ days, and the density of naïve B-cells, $B_{N}=5\times 10^{4}$ cells/mL. Changing these parameters to values appropriate to describe the blood of younger individuals can increase the protection time. Increasing $\tau_{BM}$ from 18 days to 25 days [42] has a minor effect on protection. A strong effect, however, is seen when increasing $B_{N}$ from 0.5 to $1.0\times 10^{5}$ cells/mL [58]. The higher $B_{N}$ density increases the protection time by more than 10 weeks. In this case, even following a single dose the critical titer is reached, although late, i.e., after more than 10 weeks only.

**Figure A2:**
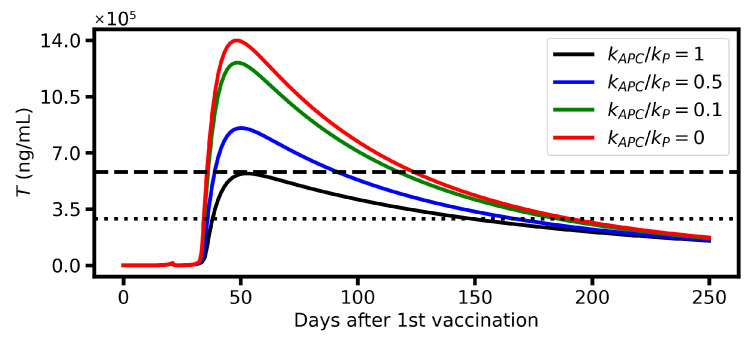
Effect of the APC activation rate on protection against virus infection. A smaller activation rate $k_{APC}$ has a small effect on the protection against the reference infection (dotted black line) only. However, it might provide protection against a mutant virus (dashed black line), even in cases where the reference immunization did not provide any protection.

While these are effects related to the variability in immune system parameters, vaccine formulation can also affect the protection level. For example, the encoded pseudo-antigen can activate susceptible cells (APCs) more or less rapidly. Smaller activation rates $k_{APC}$ thereby increase the strength of the immune response (Figure A2). This can have a strong impact, e.g., on the protection against mutant virus.

For the mutant virus, the binding strength of antibodies that have been synthesized following vaccination to the mutated antigen decreases. Accordingly, higher concentrations of pre-existing antibodies are required in order to avoid future infections. This means that for the synthesized antibodies, the critical titer, measured in equivalents of their now lower affinity, increases (Figure 7B). Accordingly, the protection time enabled by the vaccination decreases. Mutations can also affect virus replication rates $p_{V}$. In this case, the critical titer increases with increasing $p_{V}$ (Figure 7B).

### 6.3. Vaccination Timing

A parameter of the extended model that can be easily controlled to optimize the protection time is vaccination timing $t^{*}$ (Figure 7B). A maximum protection time is observed if the second dose is provided about 5 weeks after the first dose. This is in line with studies by Shioda et al. [59]. They found a better protection against SARS-CoV-2 infection for $t^{*}=26$ to 42 days (Pfizer) and $t^{*}=33$ to 49 days (Moderna) compared to FDA-recommended $t^{*}$ of 17–25 days and 24–32 days, respectively. The optimal time for the second vaccination remains stable increasing the critical titer. Thus, vaccination against mutant virus should follow the same timing.

**Figure 8:**
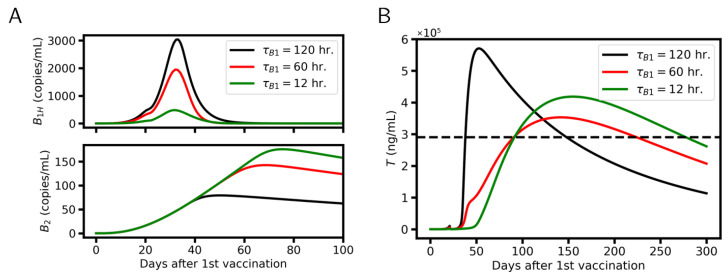
Simulation of $B_{1H}$-cell knock-down by decreasing $B_{1H}$-cell lifetime. (**A**) $B_{1H}$- (**upper row**) and $B_{2}$-cell density (**lower row**). (**B**) Acquired titer. For the short $B_{1H}$-cell lifetime, the peak of the titer moves to later times and the protection time increases from 109 to 186 days. Dashed lines refer to the critical titer $T_{c}$.

The early occurrence of high-affinity $A_{2}$ antibodies appeared as an essential problem of the vaccination process to induce long-term protection. We have shown (Figure 6) that these antibodies shutdown the GC reaction and thus impede the specification of $B_{2}$-cells. This problem does not occur during natural infection. Here, the virus reproduces itself and infects new cells. Accordingly, early-emerging high-affinity antibodies bind the virus and infected cells and do not compete with $B_{G}$-cells for antigen binding. Thus, the GC reaction remains active until the infection is stopped. In order to support this theory, we performed $B_{1H}$-cell-knock-down simulations by shortening the $B_{1H}$-cell lifetime (Figure 8). In line with our theory, the number of $B_{2}$-cells and the protection time increases. However, the peak of the titer moves to later times and protection is only reached 10 weeks after the second vaccination.

### 6.4. Sensitivity Analysis

Our results on the protection time are consistent with the observation of faster waning immunity in the elderly [60,61] and for mutant virus [62]. Next, we study the parameter sensitivity of the protection time and the amplification of antibody titer by a second dose in detail.

To identify those parameters that contribute most to the variance of these two variables, we performed a variance-based global sensitivity analysis, called the Sobol’ method or Sobol’ indices [63]. This method has been previously used in infectious disease dynamics models, including models for cholera and schistosomiasis [64]. Sobol’ indices rank the importance of each model parameter, depending on how it affects the variance of a model output. An advantage of the Sobol’ method is that it both captures the main effects of each parameter (first-order indices), as well as the interaction effects between parameters (total-order indices). Here, we provide the Total Sobol Indices (TSI) as they captures nonlinear effects when two or more parameters are varied at a time.

**Figure 9:**
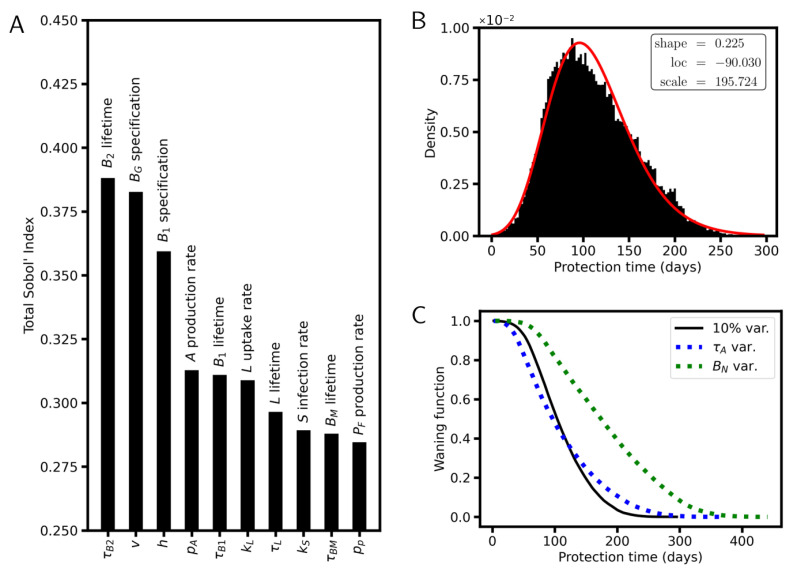
Waning immunity. (**A**) Sensitivity analysis for the protection time including all model parameters, assuming 10% variance for each. The 10 parameters of the extended model with the highest TSI are shown. (**B**) Distribution of the protection time for a population, assuming 10% variation in all model parameters. Fit (red curve): Log-normal distribution (parameters are indicated). (**C**) Waning function for the distribution of the protection time considering 10% variance and for distributions considering experimental variance for $\tau_{A}$ (30 to 90 days, [48,49]) and $B_{N}$ (5 to $15\times 10^{4}$ cells/mL).

**Figure A3:**
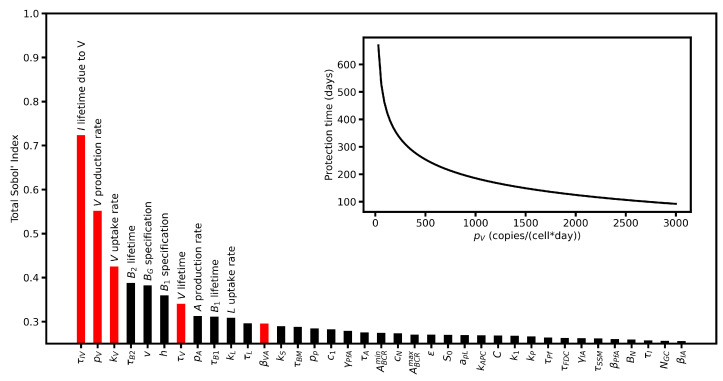
Sensitivity analysis I. TSI regarding protection time. Black: the parameters of the extended model; red: the parameters of the infection model. Insert: the protection time decreases with increasing virus replication rate $p_{V}$.

**Figure A4:**
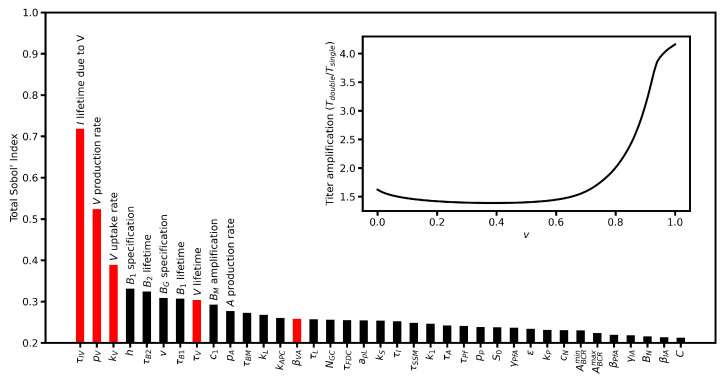
Sensitivity analysis II. TSI for titer amplification by the second compared to the first dose. Black: the parameters of the extended model; red: the parameters of the infection model. The parameters with the highest impact to the titer amplification are the lifetime of the virus infected cells $\tau_{IV}$, the virus production rate $p_{V}$ and the virus infection rate. Insert: for v>0.5, decreasing $B_{2}$ specification by increasing *v* strengthen the titer amplification.

The three parameters of the extended model that have the strongest impact on protection time are (i) the lifetime of the $B_{2}$ cells $\tau_{B2}$, (ii) the fraction of $B_{G}$ cells *v* differentiating into $B_{M}$ cells (not into $B_{2}$), and (iii) the fraction of amplifying $B_{M}$ cells *h* that differentiate into $B_{1H}$ (and do not remain $B_{M}$ cells) (Figure 9A). These are cell-intrinsic parameters. It remains unresolved whether they vary depending on the pseudo-antigen encoded by the vaccine, i.e., whether they can be modulated by selecting specific antigens. Among the parameters of the infection model used to calculate the critical titer, the parameters with the highest impact on the protection time are the lifetime of the virus-infected cells $\tau_{IV}$, the virus production rate $p_{V}$, and the virus infection rate $k_{V}$ (Figure A3). Thus, the potential protection time is most sensitive to the time of virus production, which is typically limited by type 1 IFN response [65], and to virus infection parameters. Together, these properties decide whether effective application of RNA-based vaccination is feasible and whether multiple vaccinations are required to increase the antibody titer. Protection could be improved, e.g., by modulating parameters linked to plasma cell specification. An example of this is the parameter *v*, which is the fraction of $B_{G}$-cells that differentiate into $B_{M}$- and not into $B_{2}$-cells. Here, improvement can be twofold. Smaller *v* is linked to longer protection, as the $B_{2}$-cells are the only antibody-producing cells with a lifetime of several months. However, *v* is linked to the amplification of the protection level after the second dose as well (Figure A4). A larger *v* value results in higher numbers of $B_{M}$-cells capable of differentiating into $B_{1H}$-cells and thus in higher numbers of $B_{1}$-plasma cells. This enforces the amplification of the antibody titer (Figure A4). Thus, for large *v* values, a particularly high critical titer can potentially be reached. However, the enforced amplification of the titer does reduce the protection time.

Assuming 10% variance in each parameter, a population of vaccinated individuals shows a distribution of the protection times that peaks around 100 days (Figure 9B). The distribution can be fitted by a simple log-normal distribution with a mean protection time of 111 days. One minus its cumulative distribution gives the waning function (WF) of the model (Figure 9C). These results enable future epidemiological studies and modelling to consider waning immunity based on simple functions that are derived from cell population models. The decay of the WF becomes broader if experimental variation in $\tau_{A}$ (30–90 days) or $B_{N}$ (0.5 to $1.5\times 10^{5}$ cells/mL) is considered. While symmetric variation of the antibody lifetime ($\tau_{A}$) around 60 days conserves the mean protection time, higher density of naïve B-cell ($B_{N}$), as seen in younger individuals, increases it. Actually, the mean protection time reaches about 189 days, suggesting that the protection time of younger individuals is on average 70% longer and that of elderly.

## 7. Discussion

A characteristic of RNA-based vaccination against SARS-CoV-2 is fast waning immunity. Here, we introduced a mathematical model of this vaccination method that explains this undesirable characteristic based on an early shutdown of the adaptive immune response by high-affinity antibodies. While most of the parameters of the model are derived from vaccination studies against SARS-CoV-2, we expect the principals to hold if applied to other RNA viruses.

### 7.1. Mechanisms Responsible for Waning

An essential part of the model constitutes a negative feedback loop where the antibodies synthesized by the emerging plasma cells shut down the GC reaction as well as the conversion of memory B-cells into plasma cells. Antibody-dependent control of the GC reaction has been suggested already by Zhang et al. [53]. How this control mechanism interacts with an ongoing natural infection is largely unanswered. Here, we suggest that ongoing infection consumes newly synthesized antibodies and thus enables enforced $B_{2}$ specification. In other words, the extensiveness of the infection controls whether a long-lasting protection against a secondary infection builds up. In case of RNA-based vaccination, pseudo-antigen-producing cells and the antigen itself, in most cases, vanish within a short period of time. We suggest that accordingly a quick increasing concentration of high-affinity antibodies shuts down the GC much too early to enable relevant $B_{2}$ specification, i.e., long-term protection. Thereby, pre-existing immunity, e.g., cross-reactive memory B-cells that are recalled into GCs, might affect timing [66]. We neglected these effects in the model. Recalling depends on chemokine gradients [67], which can be disturbed by specific vaccine formulations [68]. Thus, modelling effects of recalling will require vaccine-specific data on memory B-cell recruitment.

Vaccination against SARS-CoV-2 can induce long-lasting GC reactions (>30 weeks, [69]) as typically observed for virus infections [54]. This seems to argue against our thesis. However, AM against the dominant antigen might not proceed over such long periods. Experimental studies documented a permanent inflow of BN with low-affinity BCR into the GC [56]. Such behavior has been suggested to originate in competitive AM with a so-called ‘dark antigen’ [54]. The antibody feedback proposed in our model would block AM against the dominant antigen after some time and favor dark antigen-specific AM as suggested by experiments [55]. Focusing on dominant-antigen-specific AM, we did not incorporate these processes in our model. Notably, a recent publication by Mulroney et al. [70], highlights a possible source of ‘dark antigen’ in cases of vaccination against SARS-CoV-2. The authors show that incorporation of N1-methylpseudouridine into mRNA results in +1 ribosomal frameshifting. Strikingly, +1 frameshifted products from BNT162b2 vaccine mRNA translation occur after vaccination. As these products reach up to 8% of the produced protein, competitive AM might indeed be important in cases of SARS-CoV-2 vaccination.

Different processes contribute to antibody consumption. Besides direct antigen neutralization, also ADCC consumes antibodies. This process is well studied with respect to its function in SARS-CoV-2 infection [71]. Its efficacy depends on the targeted cell type. We assumed herein that infected cells are mainly APCs. In experiments, these cells show different responses to ADCC. While peripheral dendritic cells are sensitive [72], macrophages can escape ADDC [73]. Thus, it is currently not clear whether ADCC, while reducing antibody concentration, plays a substantial role in controlling numbers of ‘infected’ cells during vaccination. Similar arguments apply to ADCP and ADCD.

Our model describes the immune response to a single pseudo-antigen P, where recruitment of naïve B-cells ($B_{N}$) to GCs is limited to $c_{N}B_{N}$. What happens if vaccines are applied that contain RNA encoding different pseudo-antigens $P_{1}$ and $P_{2}$? In rodents, RNA-based vaccines combining RNA-encoding spike- and nucleocapsid-protein have been investigated [74,75]. These studies show that the response is additive. This might be a consequence of recruitment of $B_{N}$s with different BCRs. Thus, in the model, one would assume a maximum recruitment $c_{N1}BN+c_{N2}B_{N}$, with $c_{N1}$ and $c_{N2}$ being the maximum recruitment rates into $P_{1}$- and $P_{2}$-specific GCs, respectively.

If the antigens are both V-specific, a combined vaccination provides a better protection against infection with V compared to one against a single antigen. In particular, protection provided by $B_{2}$-cells secreting antibodies against $P_{1}$ will combine with that of $B_{2}$-cells secreting antibodies against $P_{2}$. Together, these antibodies could reach the critical titer and provide long-term protection. Here, one has to consider that antibodies that do not block virus entry receptors, as, those against nucleocapsid-protein do not provide a neutralization function, for example. Moreover, in mice, spike–nucleocapsid-based vaccines have been found to be associated with high-frequency lung pathology [75].

Large differences are observed in the timing of a natural infection such as that of SARS-CoV-2 [76]. According to our results, these differences can be expected to result in large differences in long-term protection against re-infection. Thus, in order to estimate protection times, models of the immune response to natural infection have to consider the timing and spread of the infection carefully. To some extent, this also applies to our model of the vaccination process. Alterations of the pool of susceptible cells induced by changing APC activation or regeneration rates affect the strength of the immune response. Thus, appropriate modelling is of particular importance for predicting protection against virus variants with reduced antigen–antibody affinity.

### 7.2. Waning Immunity in Cohorts

We have shown that the variability in immune response in individuals, originating, e.g., in different immune-cell densities, results in a broad distribution of waning rates in accordance with experimental studies [60,61]. Unfortunately, biological variances in essential parameters, such as the rates of $B_{1}$ or $B_{2}$ specification, are currently unknown. Thus, a quantitative modelling of waning functions is still not feasible. Nevertheless, our studies enable estimations of the importance of individual parameters for long-term protection. Among those showing age-related changes, the size of the compartment of naïve B-cells ($B_{N}$) appears to be important. High numbers improve the protection induced by vaccination. This suggests that comorbid conditions or repeated antigenic exposures that reduce naïve B-cell pools may impair vaccine-induced responses. Actually, HIV-infection is linked to a reduction of $B_{N}$ numbers by about 50% [77,78], i.e., similar to that seen during aging. In parallel, it is accompanied by a weaker response to SARS-CoV-2 vaccination compared to controls [79]. This also holds for the vaccination of HIV-positive individuals against other infections. Whether there is a causal link to reduced $B_{N}$-cell density remains unanswered.

We would like to emphasize that our model only explains protection against infection, i.e., its probability, but not its severity. Severity of infection might correlate with immune response [80]. Nevertheless, predicting it from acquired antibody titers alone will fail. Currently, even an estimate of the critical titer required to prevent infection with the original or derived virus strains is challenging. Virus variants with a mutated antibody-binding site or altered replication rates typically reduce the vaccine-induced immunity. Immunization against the Wuhan variant of SARS-CoV-2 was very efficient. The Omicron variant showed high unreliability regarding vaccination-induced immunization due to mutations in the receptor-binding domain of the spike protein. Changes in the BCR affinity distribution following SARS-CoV-2 evolution have been quantified [15].

Our model can consider such changes by changing antibody–BCR interaction strength. More sophisticated modelling strategies that integrate binding-site variability have been introduced recently [33]. Prediction of virus-specific protection would require a comprehensive characterization of the strain of interest, including, e.g., the distributions of virus replication rates and of the antigen–antibody affinity. Subsequent identification of model parameters from these high-dimensional data requires a dimensionality reduction, e.g., by machine learning approaches. Recently, AI-enhanced model calibration methods have widely been applied in epidemiology [81]. In parallel, sophisticated spatio-temporal models of tissue spreading are required. This process can vary even between closely related virus strains e.g., due to variable tropism [82].

Mathematical modelling of the immunity build-up and waning following RNA-based vaccination, as introduced here, aims at (i) supporting optimal timing of the vaccination process, (ii) identifying cohorts at risk for rapid waning (e.g., individuals with low naïve B-cell pools), and (iii) suggesting modifications of vaccine formulation for virus variants. Moreover, it provides a theory-based option to include proper waning into epidemiological models. Thereby, the overall objective is to help optimizing resources. Compared to AI-driven predictive models [83], the regulatory mechanisms underlying our model enable scenario analysis as exemplified in this study. The current version of our model supports the understanding of the general kinetics of the adaptive immune response. A future challenge is quantitative modelling of the immune response induced by specific vaccine formulations and the thereby achieved protection against specific virus strains. As a first step, such modelling requires a better model parametrization.

With regard to the model parameters with highest Sobol’ indices, of particular importance for this effort are data on pseudo-antigen kinetics, B-cell subset dynamics ($B_{G}$, $B_{M}$, $B_{2}$), longitudinal antibody titers (both affinity and quantity), viral replication kinetics for the circulating variant, and data on breakthrough infection timing in well-characterized cohorts.

## Figures and Tables

**Figure A1:**
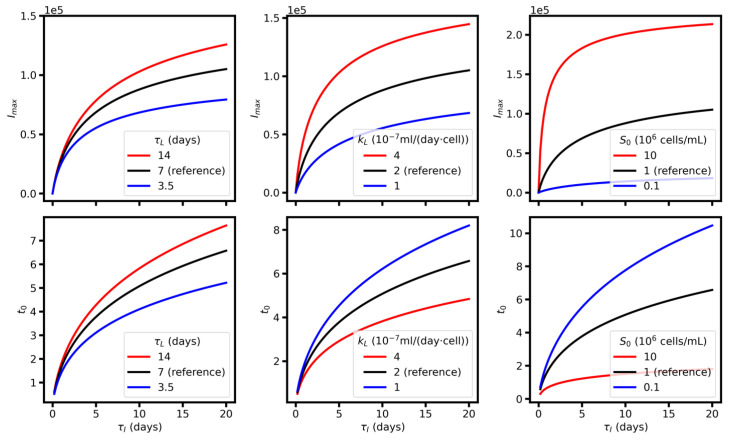
Maximum density of infected cells $I_{max}$ following vaccination. For a larger lifetime of the infected cells, $\tau_{I}$, the maximum occurs later and reaches higher densities. Similar changes are observed for larger $\tau_{L}$. Increasing $k_{L}$ and $S_{0}$, however, increases the maximum cell density but shortens the time needed to reach it.

## Data Availability

The source code for the model is available on https://github.com/jarmsmagalang/immu_buildup_waning (accessed on 12 December 2025).

## Appendix A. Analytical Solutions for the Vaccination Model

For $S=S_{0}$, the concentration of LNPs is given by:

(A1) $$L=L_{0}\exp[-(k_{L}S_{0}+\frac{1}{\tau_{L}})t].$$

Setting $k_{L}S_{0}+\left(1/\tau_{L}\right)=1/\tau_{LC}$, one obtains the density of infected cells:

(A2) $$I=I_{0}[\exp(-\frac{t}{\tau_{I}})-\exp(-\frac{t}{\tau_{LC}})]$$

with $I_{0}=\left(k_{S}S_{0}L_{0}\tau_{I}\right)\left(\left(\tau_{I}/\tau_{LC}\right)-1\right)$. The maximum density of infected cells is given by

(A3) $$I_{max}=k_{S}S_{0}L_{0}\tau_{I}x^{-\frac{x}{x-1}},$$

with $x=\tau_{I}/\tau_{LC}>1$. It is reached at $t_{0}=\left(\tau_{I}\ln x\right)/\left(x-1\right)$. Setting $k_{P}S_{0}+\left(1/\tau_{Pf}\right)=1/\tau_{PC}$, one obtains for the concentration of free-floating protein:

(A4) $$\begin{array}{cc} P_{f}= & P_{0}[\frac{\tau_{I}}{\tau_{I}-\tau_{PC}}(\exp(-\frac{t}{\tau_{I}})-\exp(-\frac{t}{\tau_{PC}}))\\ \; & -\frac{\tau_{LC}}{\tau_{LC}-\tau_{PC}}(\exp(-\frac{t}{\tau_{LC}})-\exp(-\frac{t}{\tau_{PC}}))],\;\mathrm{with}\;P_{0}=p_{P}I_{0}\tau_{PC}, \end{array}$$

and for the immune complex $P_{FDC}$ (similarly for $P_{SSM}$ but with $\tau_{SSM}$):

(A5) $$\begin{array}{cc} P_{FDC}= & \frac{k_{P}}{2}S_{0}P_{0}\tau_{FDC}[\frac{\tau_{I}^{2}}{\left(\tau_{I}-\tau_{PC}\right)\left(\tau_{I}-\tau_{FDC}\right)}(\exp(-\frac{t}{\tau_{I}})-\exp(-\frac{t}{\tau_{FDC}}))\\ \; & -\frac{\tau_{LC}^{2}}{\left(\tau_{LC}-\tau_{PC}\right)\left(\tau_{LC}-\tau_{FDC}\right)}(\exp(-\frac{t}{\tau_{LC}})-\exp(-\frac{t}{\tau_{FDC}}))\\ \; & +\frac{\tau_{PC}^{2}\left(\tau_{I}-\tau_{LC}\right)}{\left(\tau_{PC}-\tau_{I}\right)\left(\tau_{PC}-\tau_{LC}\right)\left(\tau_{PC}-\tau_{FDC}\right)}(\exp(-\frac{t}{\tau_{PC}})-\exp(-\frac{t}{\tau_{FDC}}))] \end{array}$$

## Appendix B. Details on Antibody Feedback

Activation of the GC depends on the concentrations of immune complex $P_{FDC}$ and of the antibodies $A_{1}$ and $A_{2}$. $B_{G}$ cells bind the antigen $P_{FDC}$ via their BCR. Thereby, they compete with free-floating antibodies. Assuming a competitive binding of antigen by BCRs and antibodies, classical enzyme kinetics provides the following for the concentration of the antigen–BCR complex:

(A6) $$\left[P_{FDC}B\right]=\frac{P_{FDC,GC}}{1+\frac{K_{B}}{B}+\frac{K_{B}}{K_{A}}\frac{A}{B}},$$

where $P_{FDC,GC}$ is the total concentration of $P_{FDC}$ in the GC, and $K_{B}$, $K_{A}$ the dissociation constants of BCR- and A-binding, respectively. The BCR density is given by $B=nB_{G,GC}$. Here, *n* is the number of BCR per $B_{G}$ cell [copies/cell] and $B_{G,GC}$ the concentration of $B_{G}$ cells in the GC. The concentration $\left[P_{FDC}B\right]$ divided by $B_{G,GC}$ is the number of complexes per $B_{G}$ cell [copies/cell]:

(A7) $$\frac{\left[P_{FDC}B\right]}{B_{G,GC}}\approx \frac{P_{FDC,GC}}{B_{G,GC}+\frac{K_{B}}{K_{A}}\frac{A}{n}}.$$

We assume that the activation $g_{GC}$ of the germinal center requires that a sufficient number 1/C [copies/cell] of BCRs are bound by antigen.

(A8) $$g_{GC}=1-\exp(-\frac{\left[P_{FDC}B\right]}{B_{G,GC}}C)$$

Typically, 10–20 bound BCR are required to activate a signal in B-cells. We set C=0.1. We estimate the parameters of Equations (A7) and (A8) as follows: $P_{\left(FDC,GC\right)}$ can be calculated from $P_{FDC}$ considering that the complexes are concentrated in GCs (1 GC/mL), leading to $P_{\left(FDC,GC\right)}=P_{FDC}\;\mathrm{mL}/1\;V_{GC}$. With a GC-volume of $V_{GC}=2\times 10^{-5}$ mL [37], it follows that $P_{\left(FDC,GC\right)}=5\times 10^{4}\;P_{FDC}$. The same factor applies to the concentration of $B_{G}$s: $B_{G,GC}=5\times 10^{4}\;B_{G}$. Calculating A in ng/mL, it follows that:

(A9) $$\frac{\left[P_{FDC}B\right]}{B_{G,GC}}\approx \frac{P_{FDC,GC}}{B_{G,GC}+\frac{K_{B}}{K_{A}}x\frac{A}{n}},$$

where *x* gives the number of antibodies per ng [copies/ng]. The constant $r=\left(xK_{B}\right)/\left(nK_{A}\right)$ [cells/ng] is antibody-specific. For an antibody with the same dissociation constant as the BCR follows: r=x/n. With $x=4\times 10^{9}$ copies/ng and $n=10^{5}$ copies/cell, the following applies: $r=4\times 10^{4}$ cells/ng. Thus, activation $g_{GC}$ is given by:

(A10) $$g_{GC}=1-\exp(-\frac{\left(5\times 10^{4}\right)P_{FDC}}{\left(5\times 10^{4}\right)B_{G}+\left(4\times 10^{4}\right)\frac{cells}{ng}A}C)\approx 1-\exp(-\frac{P_{FDC}}{B_{G}+\frac{cells}{ng}A}C)$$

In case of $B_{M}$ activation, these cells are concentrated in subcapsular proliferative foci (SPF) and interact with subcapsular sinus macrophages (SSM) that present interaction complexes $P_{SSM}$ [12]. We assume that SPF have to confine antigen and B-cells similar to the GC. Thus, the concentration of antigen and the density of B-cells are similarly increased in this structure. It follows that:

(A11) $$g_{BM}=1-\exp(-\frac{\left(5\times 10^{4}\right)P_{SSM}}{\left(5\times 10^{4}\right)B_{M}+\left(4\times 10^{4}\right)\frac{cells}{ng}A})\approx 1-\exp(-\frac{P_{SSM}}{B_{M}+\frac{cells}{ng}A}C)$$

Thus, activation properties are similar to the GC, but depend on the short-living interaction complex $P_{SSM}$.

Equations (A10) and (A11) describe activation functions for binding competition for antigens between BCRs and antibodies with the same dissociation constants. To consider different affinities of both, we assume:

(A12) $$A_{GC}=\frac{A_{1}A_{BCR}^{min}+A_{2}A_{BCR}^{max}}{A_{BCR}},\;\mathrm{for}\;g_{GC}$$

(A13) $$A_{BML}=\frac{A_{1}A_{BCR}^{min}+A_{2}A_{BCR}^{max}}{A_{BCR}^{min}},\;\mathrm{for}\;g_{BML}$$

(A14) $$A_{BMH}=\frac{A_{1}A_{BCR}^{min}+A_{2}A_{BCR}^{max}}{A_{BCR}^{max}},\;\mathrm{for}\;g_{BMH}.$$

The rates of cell destruction, $\beta_{IA1}$ and $\beta_{IA2}$, and the rates of antigen neutralization, $\beta_{PfA1}$ and $\beta_{PfA2}$, depend on the affinity of the antibodies as well. We assume:

(A15) $$\beta_{IA1}=\beta_{IA}A_{BCR}^{min}\;\mathrm{and}\;\beta_{IA2}=\beta_{IA}A_{BCR}^{max},$$

(A16) $$\beta_{PfA1}=\beta_{PfA}A_{BCR}^{min}\;\mathrm{and}\;\beta_{PfA2}=\beta_{PfA}A_{BCR}^{max}.$$

Values of $\beta_{IA}$ and $\beta_{PfA}$ are provided in Table A3. Similarly, we assume for the antibody consumption rates $\gamma_{IA1}$, $\gamma_{IA2}$, $\gamma_{PfA1}$ and $\gamma_{PfA2}$:

(A17) $$\gamma_{IA1}=\gamma_{IA}A_{BCR}^{min}\;\mathrm{and}\;\gamma_{IA2}=\gamma_{IA}A_{BCR}^{max},$$

(A18) $$\gamma_{PfA1}=\gamma_{PfA}A_{BCR}^{min}\;\mathrm{and}\;\gamma_{PfA2}=\gamma_{PfA}A_{BCR}^{max}.$$

Values of $\gamma_{IA}$ and $\gamma_{PfA}$ are provided in Table A3.

We set $\beta_{PfA}=\beta_{IA}=5\times 10^{-5}\mathrm{mL}/\left(\mathrm{day}\times \mathrm{ng}\right)$. Thus, at the maximum concentration of antibodies reached in our simulations (about $6\times 10^{4}$ ng/mL), the antibody-depending terms in Equation (3^#^) and Equation (4^#^) reach (−3/(day×I)) and (−3/$\mathrm{day}\times P_{f}$)), respectively. This means that all infected cells become destroyed and all free protein neutralized within 8h. The amount of antibody required for these processes and thus the parameters $\gamma_{IA}$ and $\gamma_{PfA}$ were estimated from experiments.

### Appendix B.1. ADCC Experiment

Infected cells (target cells, initial density $I_{0}$) and neutrophils are mixed with antibodies (initial concentration $A_{0}$). Infected cells fluoresce due to an integrated reporter gene. The total fluorescence of the population decreases due to ADCC by neutrophils. (time scale: 12 h). Assuming a constant neutrophil density, we calculate *I* and *A* according to:

(A19) $$\frac{dI}{dt}=\beta_{IA}AI$$

(A20) $$\frac{dA}{dt}=\gamma_{IA}AI$$

with the rate of cell destruction by ADCC, $\beta_{IA}$ [mL/(μg×day)], and the rate of antibody consumption $\gamma_{IA}$ [mL/(cell×day)].

Here, $n=\gamma_{IA}/\beta_{IA}$ [μg/cell] is the amount of antibody required per cell. The analytical solution for the remaining infected cells (*I*) is given by:

(A21) $$I=I_{0}\frac{A_{0}-nI_{0}}{A_{0}\exp(t\beta_{IA}\left(A_{0}-nI_{0}\right))-nI_{0}}.$$

For $A_{0}<nI_{0}$, one observes the following over a long period of time:

(A22) $$I=I_{0}\left(1-\frac{A_{0}}{nI_{0}}\right),$$

otherwise, I=0.

In Alpert et al. [84], they show that for HIV infection, an initial antibody concentration of $A_{0}=100$ μg/mL and an initial density of infected cells of $I_{0}=5\times 10^{4}$ cells/mL, allows 20% of the cells to survive, giving: n=2.5 ng/cell.

### Appendix B.2. Neutralization Experiment

Pseudo-virus (initial concentration $P_{f0}$) is mixed with antibodies (initial concentration $A_{0}$). The remaining capability of the mixture to infect cells that fluoresce due to an integrated reporter gene is measured (time scale: 10 min).

(A23) $$\frac{dP_{f}}{dt}=\beta_{PfA}AP_{f},$$

(A24) $$\frac{dA}{dt}=\gamma_{PfA}AP_{f},$$

with the rate of cell neutralization by ADCC, $\beta_{PfA}$ [mL/(ng×day)], and the rate of antibody consumption, $\gamma_{PfA}$ [mL/(copy×day)].

Here, $m=\gamma_{PfA}/\beta_{PfA}$ [ng/copy] is the amount of antibody required per antigen. The analytical solution for the remaining infected cells *I* is given by:

(A25) $$P_{f}=P_{f0}\frac{A_{0}-mP_{f0}}{A_{0}\exp(t\beta_{PfA}\left(A_{0}-mP_{f0}\right))-mP_{f0}}.$$

For $A_{0}<mP_{f0}$, one observes the following over a long period of time:

(A26) $$P_{f}=P_{f0}\left(1-\frac{A_{0}}{mP_{f0}}\right),$$

otherwise, $P_{f}=0$.

In Chen et al. [85], they show that the total neutralization is reached by 10% of the serum antibody level ($A_{0}=1$ μg/mL). In their assays, they use $P_{f0}=500$ TCID50. Assuming TCID50 $=4\times 10^{3}$ copies/mL [86], this gives: $P_{f0}=2\times 10^{6}$ copies/mL and $m=5\times 10^{-4}$ ng/copy.

Using the values of *n* and *m*, the parameters $\gamma_{IA}$ and $\gamma_{PfA}$ were calculated from $\beta_{IA}$ and $\beta_{PfA}$.

## Appendix C. Virus Infection Model

We assume that virus V infects a specific tissue only. The tropism of V is thereby determined by the level of expression of virus entry receptors [87]. In the following, we model an infection of the respiratory tract. We assume initial cell-free infection. Subsequent spreading depends on the site of virus release. While the apical release of virus into lumen is typically associated with local infection, basolateral release can result in systemic infections. We assume virus release into the lumen. Nevertheless, infected immune cells, as macrophages, can contribute to spreading [88]. We neglect this contribution.

### Appendix C.1. Susceptible Cells

Spreading via cell–cell contacts limits the infection rate [89]. For a non-spatial population model, we assume:

(A27) $$\frac{dS}{dt}=-k_{VI}(1-\frac{I}{I_{max}})SV+k_{2}\left(S_{0}-S\right).$$

The virus infection rate $k_{VI}$ depends on the virus variant. We set $k_{VI}=10^{-7}$ mL/(day×copy). This value is in the range that has been assumed for coronaviruses [90]. The maximum density of infected cells typically reaches 10% of $S_{0}$ [91]. Regeneration depends on lung epithelial cell proliferation.

### Appendix C.2. Infected Cells

Virus-infected cells (*I*) produce a virus until they undergo lysis or become subjects of phagocytosis [92]:

(A28) $$\frac{dI}{dt}=k_{VI}(1-\frac{1}{I_{max}}I)SV-\frac{1}{\tau_{I}}I-\left(\beta_{IA1}A_{1}+\beta_{IA2}A_{2}\right)I,$$

The lifetime of the infected cells is short. It depends on properties of the innate immune response. We set it to the time of virus reproduction, $\tau_{I}=0.5$ days [93]. $\beta_{IA1}$ and $\beta_{IA2}$ [mL/(ng×day)] are the rates of destruction of infected cells following labeling by antibodies $A_{1}$ and $A_{2}$, respectively. Values were taken from the extended model. The antibodies are present at the time of infection due to vaccination. Note that in the model, $\beta_{IA1}$ and $\beta_{IA2}$ link the tissue concentrations of cells to body concentrations of antibodies.

### Appendix C.3. Virus

Free-floating virus (*V*) can be taken up by susceptible cells that accordingly become infected, and can be degraded or neutralized by antibodies A. As *V* is shed into the lumen [94], its concentration is measured in copies per mL fluid.

(A29) $$\frac{dV}{dt}=p_{V}I-k_{V}(1-\frac{1}{I_{max}}I)SV-\frac{1}{\tau_{V}}V-\left(\beta_{VA1}A_{1}+\beta_{VA2}A_{2}\right)V,$$

Here, $p_{V}$ is the virus production rate of infected cells. They can vary between 240 and 8000 copies/(day×cell) [93]. We set $p_{V}=2500$ copies/(day×cell). Assuming that one virus infects one cells, i.e., $k_{V}/k_{VI}=1$ copy/cell, one gets: $k_{V}=10^{-7}$ mL/(day×copy). We set the virus lifetime to $\tau_{V}=2.0$ days, being at the lower bound of values found in respiratory secretions [95]. $\beta_{VA1}$ and $\beta_{VA2}$ are virus neutralization rates by antibodies $A_{1}$ and $A_{2}$, respectively.

### Appendix C.4. Analytical Solution for the Critical Titer T_c_

We first set $\beta_{IA1}A_{1}+\beta_{IA2}A_{2}=\beta_{IA}T$ and $\beta_{VA1}A_{1}+\beta_{VA2}A_{2}=\beta_{VA}T$ (see Equations (A15) and (A16)). For fast regeneration ($S=S_{0}$) and slowly evolving infection (dI/dt=0), it follows:

(A30) $$\begin{array}{cc} \frac{dV}{dt} & =p_{V}I-[\frac{k_{V}}{k_{VI}}+\frac{\frac{1}{\tau_{V}}+\beta_{AV}T}{(1+\frac{1}{I_{max}}I)k_{VI}S_{0}}](\frac{1}{\tau_{I}}+\beta_{AI}T)I\\ \; & =(p_{V}-p_{0})I. \end{array}$$

The virus population vanishes for dV/dt<0, which is observed for $p_{V}<p_{0}$. The virus production rate that can be tolerated without antibody for a starting infection (I=0) is:

(A31) $$p_{VC}=[\frac{k_{V}}{k_{VI}}+\frac{\frac{1}{\tau_{V}}}{k_{VI}S_{0}}]\frac{1}{\tau_{I}}$$

For the reference parameter set, one finds $p_{VC}=12$ copies/(day × cell), which is below reported rates. For higher $p_{V}$, antibodies are required to control the infection. The critical titer is given by:

(A32) $$\begin{array}{cc} T_{c} & =-\frac{x}{2}+\sqrt{\frac{x^{2}}{2}-y}>0,\;\mathrm{for}:\;p>p_{VC},\\ \mathrm{with}:\;\;x & =\frac{1}{\beta_{IA}\tau_{I}}+\frac{1}{\beta_{VA}}(\frac{1}{\tau_{V}}+k_{V}S_{0})>0,\\ y & =\frac{1}{\beta_{VA}\beta_{IA}}[(\frac{1}{\tau_{V}}+k_{V}S_{0})\frac{1}{\tau_{I}}-pk_{VI}S_{0}]<0. \end{array}$$

$T_{c}$ depends on parameters of the virus infection model. We included these dependencies in our calculations of the parameter sensitivity for the protection time (Figure A3). The titer amplification by the second dose (Figure A4) does not depend on $T_{c}$.

## Appendix D. Reference Parameter Sets

The following tables provide the reference parameter sets of the combined model (Table A1, Table A2, Table A3 and Table A4) and the model of virus infection (Table A5). Each considers results from a broad set of experimental techniques. Among them are RNA lifetime assays (linkage duplex ddPCR [19]), protein secretion and decay assays (isotope dilution mass spectrometry [28], disposable photonics [29]) and functional antibody assays [85]. Moreover, selected GC parameters were taken from sophisticated agent-based modelling studies [33]. For a detailed description of experiments and simulation studies, we refer to the respective references.

**Table A1:** Vaccination model.

Parameter	Description	Value	Reference
$k_{L}$	Uptake rate of *L*	$2\times 10^{-7}$ mL/day/cell	[19]
$\tau_{L}$	Lifetime of *L*	7 days	[19]
$k_{S}$	Infection rate	$2\times 10^{-7}$ mL/day/cell	[19]
$\tau_{I}$	Lifetime of *I*	4 days	[25]
$k_{1}$	Recruitment rate of *S*	$7.1\times 10^{-2}$ /day	Set
$S_{0}$	*S*-pool density	$10^{6}$ cells/mL	[23]
$p_{P}$	Protein production rate	25 copies/day/cell	[28]
$k_{p}$	Internalization rate of $P_{f}$	$10^{-7}$ mL/day/copy	See text
$k_{APC}$	Activation rate of APC	$10^{-7}$ mL/day/copy	See text
$\tau_{Pf},\tau_{PSSM}$	Lifetime of $P_{f}$ and $P_{SSM}$	2 days	[29]
$\tau_{PFDC}$	Lifetime of $P_{FDC}$	20 days	[32]

**Table A2:** Response models.

Parameter	Description	Value	Reference
$B_{N}$	$B_{N}$ density	$5\times 10^{4}$ cells/mL	See text
$N_{GC}$	Number of productive GCs	1 /mL	See text
$c_{N}$	Maximum $B_{N}$ recruitment rate	$10^{-3}$/day	[33]
$a_{pL}$	GC apoptosis rate	0.5/day	Set
ϵ	Rate of $B_{G}$ leaving GC	0.05/day	[33]
$A_{BCR}^{min}$	Minimum $A_{BCR}$	1	Set
$A_{BCR}^{max}$	Maximum $A_{BCR}$	30	Set
*v*	Fraction of $B_{G}$ to $B_{MH}$	0.9	Set
$c_{1}$	Maximum $B_{M}$ amplification rate	0.6/day	[46]
*h*	Fraction of $B_{M}$ to $B_{1}$	0.6	Set
$\tau_{BM}$	Lifetime of $B_{M}$	18 days	[42]
$\tau_{B1}$	Lifetime of $B_{1}$	5 days	[47]
$\tau_{B2}$	Lifetime of $B_{2}$	180 days	[45]
$p_{A}$	Antibody production rate	2 ng/day/cell	[50]
$\tau_{A}$	Antibody lifetime	60 days	[48,49]

**Table A3:** Nonlinear terms and activation functions derived in Appendix B.

Parameter	Description	Value
*C*	Scaling rate	0.1 cells/copy
$\beta_{IA}$	Cell destruction rate	$5\times 10^{-5}$ mL/day/ng
$\beta_{PfA}$	Neutralization rate	$5\times 10^{-5}$ mL/day/ng
$\gamma_{IA}$	Antibody consumption of *I*	$2.5\times \beta_{IA}$ ng/cell
$\gamma_{PfA}$	Antibody consumption of $P_{f}$	$5\times 10^{-4}\times \beta_{APf}$ ng/copy

**Table A4:** Initial densities of cells, antigens, and antibodies.

Population	Initial Density
*L*	$2.5\times 10^{5}$ copies/mL [19]
*S*	$S_{0}$
$I,B_{G},B_{M},B_{1},B_{2}$	0
$P_{f},P_{FDC},P_{SSM}$	0
$A_{1},A_{2}$	0

**Table A5:** Virus infection model: Parameters defining $T_{c}$. The rates of cell destruction, $\beta_{IA1}$ and $\beta_{IA2}$ and the rates of antigen neutralization $\beta_{VA1}$ and $\beta_{VA2}$ are taken from the response model ($\beta_{VA1}=\beta_{PfA1}$ and $\beta_{VA2}=\beta_{PfA2}$).

Parameter	Description	Value	Reference
$k_{VI}$	Cell infection rate	$10^{-7}$ mL/days/copy	Set
$\tau_{I}$	Lifetime of infected cells	0.5 days	[93]
$S_{0}$	*S*-pool density	$10^{6}$ cells/mL	Set
$k_{V}$	Virus internalization rate	$10^{-7}$ mL/days/cell	Set
$p_{V}$	Virus production rate	2500 copies/day/cell	[93]
$\tau_{V}$	Virus lifetime	2 days	[95]
